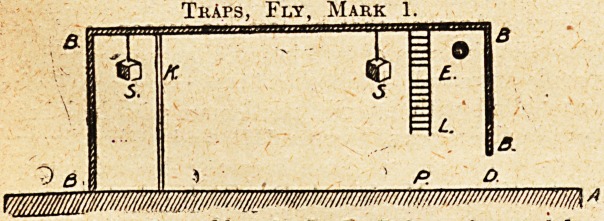# The Great War: IX. Flies and Disease (concluded)

**Published:** 1918-06-22

**Authors:** 


### THE GREAT WAR.

### IX.—Flies and Disease (*Concluded from page 202*).

### THE PREVENTION OF FLIES.

The prevention of flies on the Western Front has been a laborious and uphill task. Ignorance, scepticism, and indifference, both on the part of the French inhabitants and the British Army, had to be overcome by gradual and persistent education and the creation of an atmosphere of good will. The essential initial measure of the removal of litter regularly to selected dumps at some distance from habitations was particularly difficult on the part of the French, the *fumiers* had existed from time immemorial, and the national system of intensive cultivation was dependent on the ripe fertilisers which where obtained from them. The sheep-pens also were cleaned out only at manuring time, the deep layer of stored litter serving the double purpose of fermentation and a warm bed for the sheep at shearing time.

According to French law all litter is the property of the farmer or landlord, the hiring of a stable or farm does not entitle the lessee to dispose of the litter of his own animals as he may wish.

The French Government were approached with a proposal for the introduction of compulsory removal of litter from the vicinity of dwellings, but without success. The Prefects of provinces, however, were sympathetic, and notices, explanatory and persuasive, were circulated through the *sous* Prefects and Mayors which resulted in a more or less amenable attitude on the part of the townspeople and villagers, who thereafter allowed their cherished *fumiers* to be emptied regularly.

There was less difficulty with the Army. Orders and memoranda on the subject were in constant circulation from General Headquarters, and there was more or less disciplinary control, but it was only quite recently that a high state of efficiency was reached, and this coincided with the higher development of the Sanitary Sections, which selected and marked suitable dumping sites and the roads leading to them, and ensured by frequent inspections and assistance that they were properly used.

### CONSTANT REMOVAL OF LITTER THE KEY TO PREVENTION.

The mere removal of the litter to a distance of 500 to 1,000 yards to the leeward of dwellings had an enormous effect on the prevalence of flies in billets, but the latest development of the formation and treatment of dumps has still further reduced the numbers. The litter was at first deposited in cartloads in such a manner as gave the least trouble, with the result that it was spread over a large area in small heaps, which presented favourable conditions for breeding; then it was collected into conical mounds, and finally, and most successfully, in a continuous stack about 4 feet by 4 feet well rammed on the surface, a plan which offered the least surface for hatching and the most convenient dimensions for treatment. Frequent removal has an important bearing on prevention—theoretically, weekly removal should suffice; but in practice the results were not entirely satisfactory, and it was not until daily removal was enforced that the maximum results were attained.

The treatment of dumps has been an object of continuous research and experiment; attempts have been made to kill the larvæ and pupæ by the application of poisons in sprays or powders, to destroy them by the exploitation of the natural heat generated by fermentation, and to kill them by the artificial production of parasitic disease. The poisonous solutions used include sulphate of iron, 20 per cent.; slaked lime, 20 per cent.; miscible oil, 5 per cent.; cresol, 5 per cent. (one gallon to 3 square feet); and "C" solution. Of these "C" solution in full strength is the best; it is an arsenical preparation of oily consistence, the exact composition of which was not disclosed. Efficiently applied it kills eggs, larvæ, pupæ, and imagos.

Of solid applications, borax 1¼ lb. per eight bushels destroys 99 per cent. of larvæ, but it is expensive and unobtainable in sufficient quantity. A covering of green oil and earth, of a strength of 1 in 40, is also reliable, but impracticable on a large scale. The destruction of newly-born flies by the induction of epidemic disease is at present in the experimental stage. Hopes are, however, entertained that some day cultivations of *Empusa muscæ*, the parasitic fungus of the fly, will be available for its wholesale destruction. The objections to most of these artificial means of destruction are their adverse effect on manurial value, their poisonous nature, and the cost of manufacture, transport, and labour.

The destruction of manure or the addition to it of any substance which diminishes its value as a fertiliser is wasteful, and the growing tendency is to search for and develop more natural means of treatment. The utilisation of the heat of fermentation promises well; the stacks are covered with old tarpaulins, with sacks in sufficiently thick layers for the prevention of radiation, or with a layer of earth sufficiently deep to answer this purpose. During the summer, fermentation is completed in about a week, so that after that period movable coverings can be used on fresh parts of the stacks. The larvæ collect and pupate immediately under the coverings. Few imagos, however, emerge, and those which do, are immature, and weak.

Under circumstances in which prevention is impracticable, innumerable methods of trapping and poisoning are employed. Fly-papers, poisonous or "tanglefoot"; formalin, a tablespoonful to a pint of water, exposed in a plate with a piece of bread for the flies to settle upon; and various kinds of traps. All of these have their peculiar objections; the least objectionable being the common wire-gauze balloon trap, and the best bait, equal parts of casein, brown sugar, and water allowed to ferment for twenty-four hours before use; or, failing this, a mixture of beer and brown sugar.

As an indication of the growing interest taken by the "Staff" in the fly crusade we reproduce an amusing circular on the subject emanating from a high authority.

*In reply quote this number:* G.H.Q.,
AMD/2/SL/LY. 21/8/17.

### CIRCULAR MEMORANDUM.

#### EXTERMINATION OF FLIES.

Owing to the prevalence of flies, life is becoming quite intolerable out of the trenches. It is, therefore, considered desirable to in every way endeavour to as far as possible exterminate these pests. To this end it was suggested some time back that fly-papers should be issued

☛ Previous articles appeared on Feb. 9, 23, March 9, 23, April 6, 20, May 4, 25, and June 8, p. 201.

**THE GREAT WAR**—*(continued).*
to the troops, but, owing to the difficulty experienced in deciding on the scale of issue (*e.g.*, one Division suggested a scale of papers, fly, per man, per day, one; whilst another Division suggested papers, fly, per fly, per day, one), and of taking a census of the flies on any particular day, the suggestion has died a natural death, whilst flies continue to prosper. In the absence of any centralised system of dealing with this important subject units and formations must make every endeavour to compete with the fly question, and must use their own discretion as to the best methods of doing so, but in the meantime some suggestions for an improvised trap are put forward on p. 250 for the benefit of all concerned.

### AN IMPROVISED FLY "TRAP"—(*see p.* 248).

The fly enters at D, and seeing the sugar at K, hurries across to it; being frustrated in its designs by the screen, it turns away, and catching sight of the sugar at E, crosses the roof, struggling to reach the succulent morsel. Presently it reaches the ladder, and with a shout of delight commences a hurried descent, keeping its eyes fixed on the sugar; at the point E *there is a rung missing*! The fly in its hurry misses its footing and falls to the ground, where it breaks its neck on the marble slab at the point P.

Traps, Fly, Mark 1.

A is a slab of marble. B, B, B, B is an inverted box with a door, D. L is a ladder, and S, S pieces of sugar suspended by strings; K is a glass screen.

(Sd.) A. Maggot,
Major-General A.Q.M.G.

## Figures and Tables

**Figure f1:**